# Differential Cytokine Profiles upon Comparing Selective *versus* Classic Glucocorticoid Receptor Modulation in Human Peripheral Blood Mononuclear Cells and Inferior Turbinate Tissue

**DOI:** 10.1371/journal.pone.0123068

**Published:** 2015-04-13

**Authors:** Ilse M. Beck, Koen Van Crombruggen, Gabriele Holtappels, François Daubeuf, Nelly Frossard, Claus Bachert, Karolien De Bosscher

**Affiliations:** 1 Laboratory of Experimental Cancer Research (LECR), Department of Radiation Oncology & Experimental Cancer Research, Ghent University, Gent, Belgium; 2 Upper Airway Research Laboratory (URL), Ghent University Hospital, Ghent, Belgium; 3 Laboratoire d'Innovation Thérapeutique, Unité Mixte de Recherche 7200, Centre National de la Recherche Scientifique-Université de Strasbourg, Faculté de Pharmacie, Illkirch, France; 4 Division of ENT Diseases, Clintec, Karolinska Institute, Stockholm, Sweden; 5 Receptor Research Laboratories, Nuclear Receptor Lab (NRL), VIB Department of Medical Protein Research, Ghent University, Gent, Belgium; University of San Francisco, UNITED STATES

## Abstract

**Background:**

Glucocorticoid Receptor agonists, particularly classic glucocorticoids, are the mainstay among treatment protocols for various chronic inflammatory disorders, including nasal disease. To steer away from steroid-induced side effects, novel GR modulators exhibiting a more favorable therapeutic profile remain actively sought after. Currently, the impact of 2-(4-acetoxyphenyl)-2-chloro-N-methylethylammonium chloride a plant-derived selective glucocorticoid receptor modulator named compound A, on cytokine production in *ex vivo* human immune cells and tissue has scarcely been evaluated.

**Methods and Results:**

The current study aimed to investigate the effect of a classic glucocorticoid versus compound A on cytokine and inflammatory mediator production after stimulation with *Staphylococcus aureus*–derived enterotoxin B protein in peripheral blood mononuclear cells (PBMCs) as well as in inferior nasal turbinate tissue. To this end, tissue fragments were stimulated with RPMI (negative control) or Staphylococcus aureus–derived enterotoxin B protein for 24 hours, in presence of solvent, or the glucocorticoid methylprednisolone or compound A at various concentrations. Supernatants were measured via multiplex for pro-inflammatory cytokines (IL-1β, TNFα) and T-cell- and subset-related cytokines (IFN-γ, IL-2, IL-5, IL-6, IL-10, and IL-17). In concordance with the previously described stimulatory role of superantigens in the development of nasal polyposis, a 24h *Staphylococcus aureus*–derived enterotoxin B protein stimulation induced a significant increase of IL-2, IL-1β, TNF-α, and IL-17 in PBMCs and in inferior turbinates and of IL-5 and IFN-γ in PBMCs.

**Conclusion:**

Notwithstanding some differences in amplitude, the overall cytokine responses to methylprednisolone and compound A were relatively similar, pointing to a conserved and common mechanism in cytokine transrepression and anti-inflammatory actions of these GR modulators. Furthermore, these results provide evidence that selective glucocorticoid receptor modulator-mediated manipulation of the glucocorticoid receptor in human tissues, supports its anti-inflammatory potential.

## Introduction

Inflammation involves a systemic immune response of tissues to a plethora of harmful stimuli—such as bacterial lipopolysaccharides, tumor necrosis factor (TNF)α, irradiation or viral infection—and is characterized by an activator protein-1 (AP-1)- and/or nuclear factor κB (NF-κB)-mediated production of several cytokines and chemokines. Both transcription factors are ubiquitously expressed and form homo- and heterodimers. Whereas AP-1 is both nuclear and cytoplasmic, the prototypical NF-κB heterodimer p65-p50 resides mostly in the cytoplasm of unstimulated cells, with its nuclear localization signal shielded by the NF-κB-binding inhibitor of NF-κB (IκB). Upon exposure to a stressor, such as TNFα, the activated IκB kinase (IKK) complex phosphorylates IκB, resulting in the subsequent ubiquitination and proteasomal degradation of this protein. As such, activated and post-translationally modified NF-κB is free to travel to the nucleus and activate the gene promoters of multiple pro-inflammatory genes via binding to its specific recognition sites and mounting an active enhanceosome [1–3]. These AP-1- and NF-κB-enhanced genes are involved in immune responses and code for cytokines, e.g.interleukin-6 (IL-6), IL-8, IL-1, enzymes, e.g. iNOS and COX-2, and adhesion molecules, e.g. ICAM and VCAM [4,5].

Exemplary, inflammation-based chronic upper airway diseases are common and disabling afflictions [6,7] for which the current first choice treatments constitute anti-histamines and topical glucocorticoids [8,9]. Allergic rhinitis, representing an inflammation of the nasal mucosa to allergens after a sensitization process, involves histamine, leucotriene and prostaglandin release by mast cells and cytokine production by T helper (Th)2 cells. Additionally, acute post-viral and chronic rhinosinusitis, with or without nasal polyps, classify as an inflammatory reaction by Th1, Th2 or Th17 cells and implicate extensive pro-inflammatory cytokine release processes [8,9].

Glucocorticoids play a role in a number of biological processes, including development, differentiation, metabolism and homeostasis and stress control. In that respect, glucocorticoids are used as effective anti-inflammatory therapeutics in a variety of inflammation-based afflictions, including allergic rhinitis and nasal polyposis [8–12]. These steroidal glucocorticoids can bind to their cognate glucocorticoid receptor (GR, NR3C1). This receptor, and member of the nuclear receptor family, comprises a variable N-terminal activation domain, and an evolutionary conserved DNA-binding domain, hinge region and ligand binding domain, the latter of which contains yet a second activation domain [13]. Mechanistically, naive GR molecules reside in a chaperoning complex in the cytoplasm, while a ligand-activated GR translocates into the nucleus resulting in specific gene transcription and repression of gene expression. Besides other gene-activating mechanisms, the classic binding of a ligand-activated GR dimer to a palindromic glucocorticoid-responsive element (GRE) constitutes transactivation. Conversely, the prototypical transrepression mechanism is characterized by binding of GR to another DNA-bound transcription factor, such as NF-κB or AP-1 [13,14]. Alternatively, the GR can also inhibit gene expression via a negative GRE (nGRE) featuring direct DNA binding of the GR [15]. Different glucocorticoids can have different relative receptor affinities and display different pharmacokinetic parameters, ultimately affecting their anti-inflammatory activity [16]. Moreover, the use of glucocorticoids is burdened by a detrimental side effect profile, predominately but not exclusively associated with GR transactivation [17,18]. The adverse effects along with the occurrence of glucocorticoid insensitive patients [19] continuously drive the search for more selective GR modulators with a comparable anti-inflammatory or transrepressing power and with an overall improved therapeutic index. In various reports, compound A (CpdA), a stabile analogue of the hydroxy-phenyl aziridine precursor found in the Namibian shrub *Salsola tuberculatiformis* Botschantzev [20], has shown GR-dependent anti-inflammatory actions with reduced side effects, both *in vitro* and *in vivo* [21–25]. Indeed, this plant-derived compound A, i.e. 2-(4-acetoxyphenyl)-2-chloro-N-methyl-ethylammonium chloride, can bind to GR and allows GR-mediated transrepression of various cytokines and chemokines via an inhibition of NF-κB activity [21,23]. However, as compound A actively drives GR to a monomer formation and does not mediate GR Ser211 phosphorylation, compound A does not empower classic GRE transactivation mechanisms [21–23,26].

Since almost all currently published reports on compound A feature *in vitro* or murine *in vivo* data and since pathophysiological responses are still best analyzed in human subjects or at least primary cells, we set out to investigate how this selective GR modulator impacts human cells and tissues, with regard to the secretion of inflammation-regulating cytokines and the possible induction of cell toxicity. To this end, we studied *ex vivo* human PBMCs and an *ex vivo* human model for challenged nasal inferior turbinate tissue. We measured cytokines derived from different T helper cells as outcome parameters. To analyze the potential clinical applicability of selective GR modulation, exemplified by compound A, we used the bacterial enterotoxin *Staphylococcus aureus* enterotoxin B (SEB) to induce cytokine production in these tissues and cells, as an established model previously used to investigate human nasal polyposis [27].

## Material and Methods

### Patients

Nasal tissue was obtained from 9 patients (mean age, 40.7 years; range, 16–62 years; 5 male and 4 female) undergoing septal surgery and/or turbinotomy because of nasal obstruction, a routine sinus surgery at the Department of Oto-rhino-laryngology of the Ghent University Hospital. Additional PBMCs were obtained from 6 patients (mean age, 34.0 years; range, 27–41 years; 1 male and 5 female). The ethical committee of the Ghent University Hospital approved the study (2004/334), and all patients gave their written informed consent before inclusion in the study. On behalf of the minors in the study, written informed consent was obtained from the next of kin, caretaker, or guardian. None of the patients received intranasal corticosteroids, anti-histamines, anti-leukotrienes, oral or intranasal decongestants, or intranasal anti-cholinergics within 1 week before surgery, and none of the subjects received oral and/or intramuscular corticosteroids within 4 weeks before surgery. For female subjects, pregnancy or lactation was excluded.

### Mechanical disruption and stimulation of human nasal tissue

Preparation of human inferior turbinate tissue was performed, essentially as described [28]. In short, human nasal tissue was cut in RPMI1640 tissue culture medium (Sigma-Aldrich, Belgium), complemented with 2mM L-Glutamine (Invitrogen, Belgium), 50 IU/ml penicillin, 50mg/ml streptomycin (Invitrogen) and 0.1% BSA (Sigma-Aldrich). Subsequently these pieces were passed through a mesh to achieve comparable sized fragments (±0.9mm^3^). After 1h equilibration, the obtained tissue fragments were washed with fresh culture medium, weighed and resuspended into 48-well plates (BD Falcon; VWR International, Belgium) as 0.04g/ml in 0.5 ml RPMI1640 tissue culture medium, prepared as above. Tissue suspensions were pre-incubated with either solvent, methylprednisolone (MP) (ranging from 10^-4^M to 10^-11^M) or compound A (ranging from 10^-4^M to 10^-11^M) for 1 hour at 37°C and 5% CO_2_. Ensuing, tissue fragments were stimulated with 0.5 μg/ml (final concentration, fc) *Staphylococcus aureus* enterotoxin B (SEB, Sigma-Aldrich) for 24 hours. The SEB solvent PBS served as a negative control.

### Peripheral blood mononuclear cell (PBMC) analysis

Peripheral blood mononuclear cells (PBMCs) were isolated from anti-coagulated (using EDTA) human blood by density gradient centrifugation over Ficoll Paque (GE Healthcare) and consist mainly of monocytes, T cells and B cells and smaller amounts of NK cells and dendritic cells of both myeloid and plasmacytoid origin. PBMCs of 10 donors were pre-incubated with either solvent, methylprednisololone (ranging from 10^-7^M to 10^-5^M) or compound A (ranging from 10^-7^M to 10^-5^M) for 1 hour at 37°C and 5% CO_2_. Ensuing, PBMCs were exposed to either tissue culture medium or SEB (Sigma-Aldrich) at 0.5μg/ml (fc) for 24 hours. An additional pre-incubation step with RU486 (20μM) (Sigma-Aldrich) was included for particular settings in an experiment, as indicated in the figure legend.

### Cytokine production analysis

Supernatants of inferior turbinate tissue and PBMC solutions were separated by centrifugation; aliquots of the supernatants were snap frozen and stored immediately at -20°C until analysis of cytokines. Concentrations of IL-1β, IL-2, IL-5, IL-6, IL-10 and IL-17, TNFα and/or interferon-γ (IFN-γ) (detection limits 0.6 to 7.8 pg/ml) were measured using commercially available Fluorokine MAP Human Cytokine Kits by using the Fluorokine MAP Human Base Kit A (R&D Systems, MN, USA) following the instructions of the manufacturer, on a Bio-Plex 200 Array Reader (Bio-Rad, Hercules, CA, USA).

### Cell viability analyses

To assess possible cell damage and toxicity effects of the used compounds, we used the commercial QuantiChrom Lactate Dehydrogenase Kit (BioAssays Systems, Hayward, CA, USA) for a colorimetric kinetic determination of lactate dehydrogenase (LDH) activity. The analysis was performed according to the manufacturer’s instructions. The culture media from PBMCs, stimulated as indicated in the figure legends, were assessed for released LDH, with a detection limit from 2 IU/L up to 200 IU/L. Cell apoptosis was assessed by means of an annexin V FITC assay kit (Cayman Chemical) via FACS analysis gated on the lymphocytes. Propidium iodide served as a marker of cell death in this assay.

### Statistical analyses

Results are shown +/- standard error. Statistical analysis of the cytokine production analyses were performed using a Wilcoxon matched-pairs singed-ranks test for paired comparisons corrected for multiple comparisons. The results from the LDH analysis were statistically analyzed using a Friedman test. The results for the annexin V binding assay were statistically analyzed using a two-way ANOVA with Bonferroni post-tests. In all assays, *P* values below 0.05 were considered to indicate a statistically significant difference.

## Results

### The impact of compound A and methylprednisolone on Th1 cytokines

**Fig 1:**
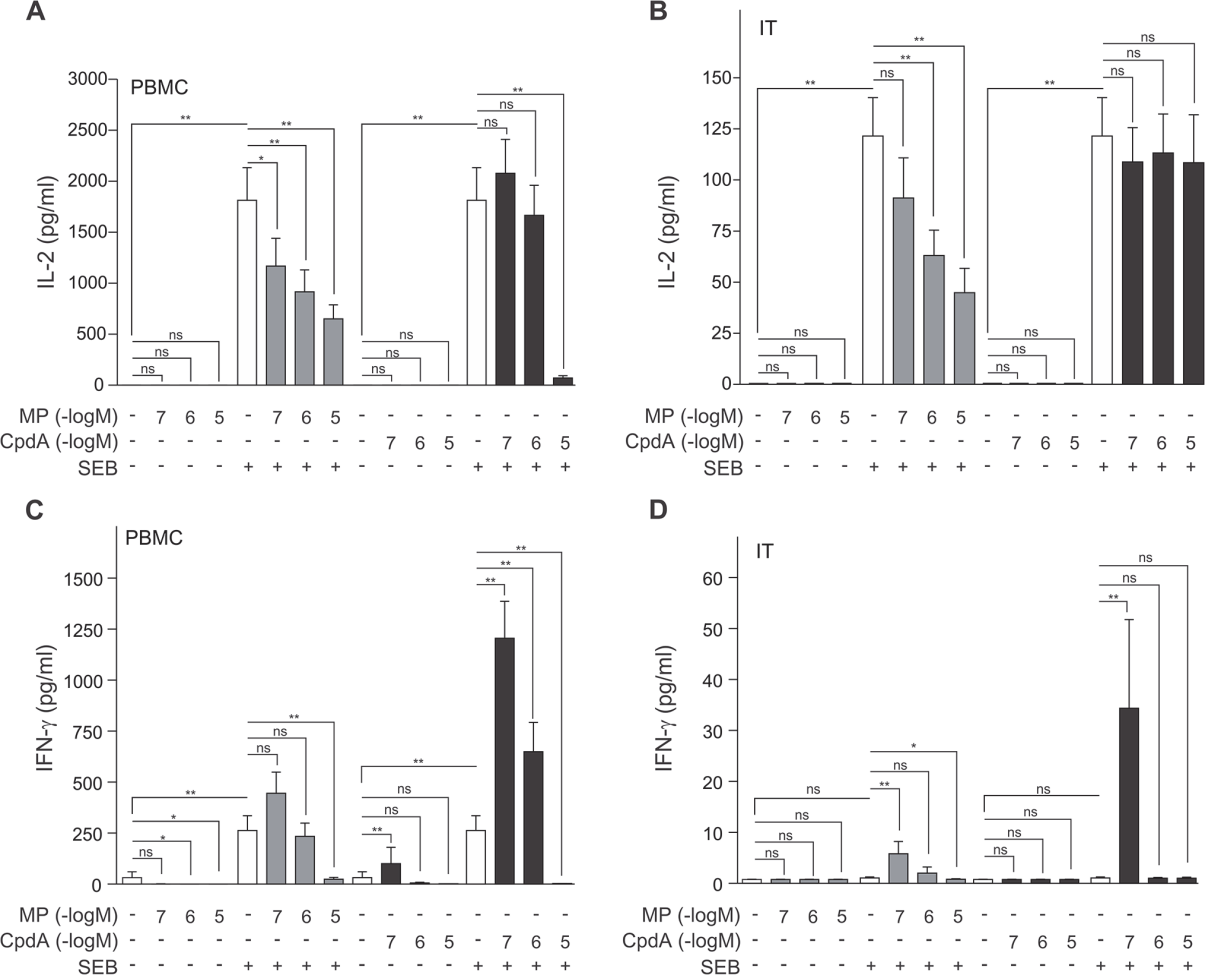
Methylprednisolone and compound A inhibit SEB-induced IL-2 and IFN-γ production with a different and tissue-dependent sensitivity. *(A*,*C)* PBMC cells and *(B*,*D)* processed nasal inferior turbinate tissues (IT) were treated with methylprednisolone (MP) (0.1μM, 1μM or 10μM) or compound A (CpdA) (0.1μM, 1μM or 10μM) for 1h, followed by a 24h incubation with SEB (0.5μg/ml). Cell culture media were analyzed for the presence of IL-2 *(A*,*B)* or IFN-γ *(C*,*D)*. Averaged results of 10 (PBMC) or 9 (IT) patient samples are shown ± SEM. Statistical analysis was performed using a Wilcoxon matched-pairs signed-rank test to analyze significance of select condition to condition comparisons. ns, not significant; **, *P*<0.01.

To investigate how the selective GR modulator compound A impacts human cells and tissues, we treated *ex vivo* human PBMCs and an *ex vivo* human model for challenged nasal inferior turbinate tissue with the bacterial enterotoxin *Staphylococcus aureus* enterotoxin B (SEB), preceded with a treatment with solvent, compound A (CpdA) or methylprednisolone (MP) in gradually increasing concentrations. Upon assaying the secreted protein levels of Th1 cytokines IL-2 and IFN-γ, it was clear that upon SEB stimulation the IL-2 production augments in both PBMCs and inferior turbinate tissue (Fig 1A and 1B), whilst the IFN-γ production is enhanced only in PBMCs and not inferior turbinate tissue (Fig 1C and 1D). Considering IL-2, we show that MP treatment results in a concentration-dependent decrease in IL-2 production in both PBMCs and inferior turbinate tissue (Fig 1A and 1B), while only the maximal concentration of compound A (10μM) is capable of significantly, but forcefully, repressing IL-2, and only in PBMCs (Fig 1A). Furthermore, we discovered that in PBMCs and inferior turbinate tissue a low concentration of compound A (0.1μM) could synergistically elevate the already SEB-stimulated IFN-γ production (Fig 1C and 1D). However, a higher concentration of compound A (10μM) antagonistically imposed a strong repression on INF-γ production in these PMBCs (Fig 1C). A similar profile, though less pronounced is also observed for SEB- and MP-treated inferior turbinate tissue (Fig 1D). Although this stimulatory trend appears also in SEB-stimulated MP-treated PBMCs, the response is not pronounced enough to reach significance, but MP at 10μM does repress SEB-stimulated IFN-γ production in PMBCs (Fig 1C). To summarize, both compound A and MP can repress IL-2 and IFN-γ production, while lower concentrations of compound A (0.1μM, 1μM) can enhance the secreted levels of IFN-γ from PBMCs.

### The impact of compound A and methylprednisolone on Th2 cytokines

**Fig 2:**
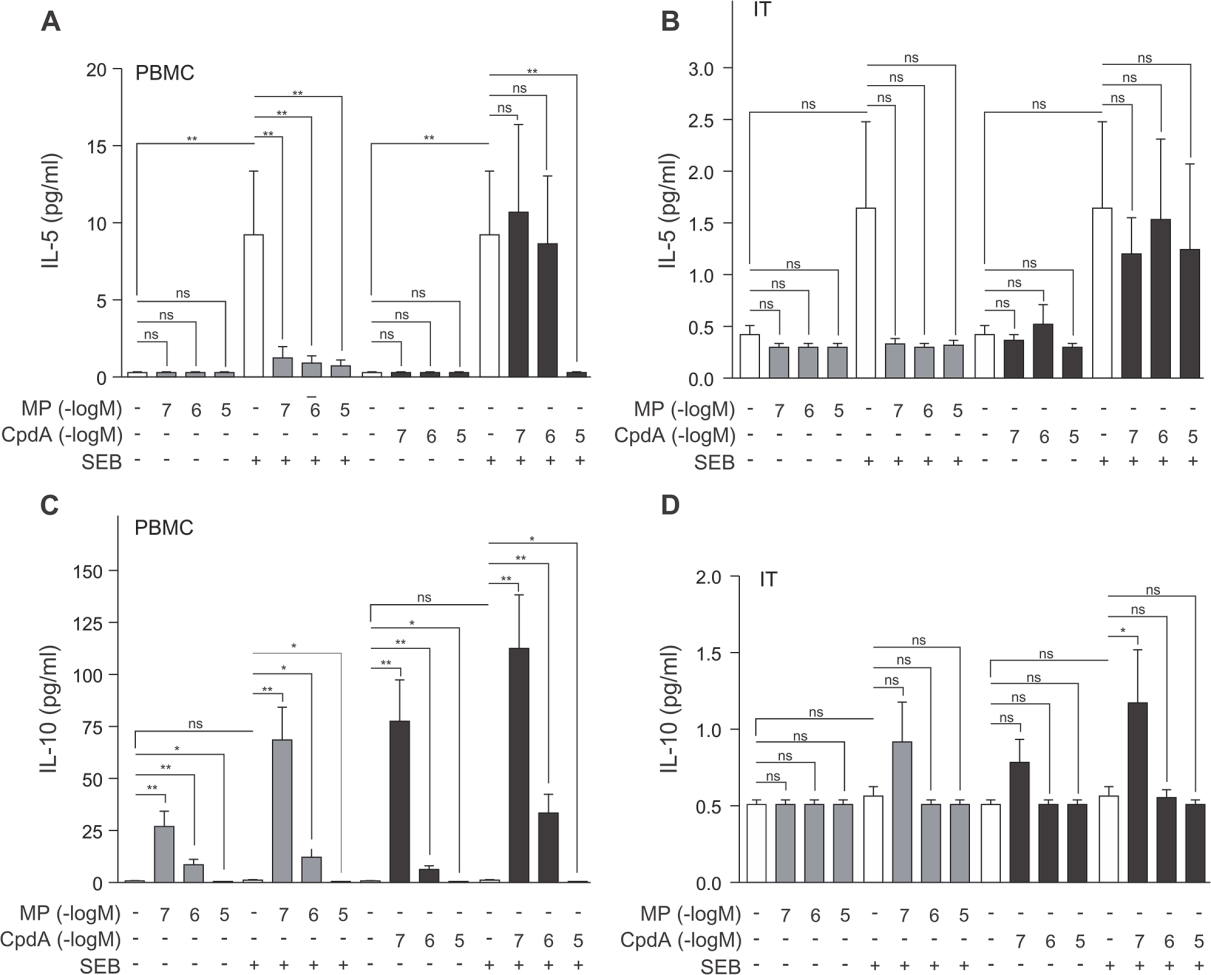
Methylprednisolone or compound A concentration-dependently impacts IL-10 production in PBMCs, while these compounds inhibit SEB-induced IL-5 production with a different and tissue-dependent sensitivity. *(A*,*C)* PBMC cells and *(B*,*D)* processed nasal inferior turbinate tissues (IT) were treated with methylprednisolone (MP) (0.1μM, 1μM or 10μM) or compound A (CpdA) (0.1μM, 1μM or 10μM) for 1h, followed by a 24h incubation with SEB (0.5μg/ml). Cell culture media were analyzed for the presence of IL-5 *(A*,*B)* or IL-10 *(C*,*D)*. Averaged results of 10 (PBMC) or 9 (IT) patient samples are shown ± SEM. Statistical analysis was performed using a Wilcoxon matched-pairs signed-rank test to analyze significance of select condition to condition comparisons. ns, not significant; *, *P*<0.05; **, *P*<0.01.

To analyze how Th2 cytokines would respond when exposed to compound A, we measured the production of IL-5, and IL-10 in the experimental setting as detailed above. The exposure of PBMCs to SEB results in an increase in IL-5 production (Fig 2A). While SEB could also induce a positive trend in IL-5 production in inferior turbinate tissue, this trend does not reach significance (Fig 2B). Our results further show that in PBMCs MP can significantly repress IL-5 secretion, while compound A can only achieve this at a higher concentration (10μM) (Fig 2A). Compound A is, however, not able to repress IL-5 production in inferior turbinate tissue (Fig 2D).

Since the cytokine IL-10 is produced by Th2 cells, but capable of inhibiting Th1 activity, we were also interested in how our selective GR agonist would affect its production. Of note, IL-10 can also be secreted by regulatory T cells. We observed that SEB is unable to significantly elevate the IL-10 levels in PBMCs (Fig 2C). Surprisingly, we observed an inverse concentration gradient for MP stimulation of IL-10 in which MP 0.1μM and MP 1μM cause a steep increase in PBMC IL-10 production, while MP 10μM actually modestly decreases SEB-stimulated IL-10 production (Fig 2C). In a similar trend, a treatment with compound A also brings about an inverse concentration gradient stimulation of IL-10 secretion in PBMCs. This compound A (0.1μM)-augmented IL-10 production is even more pronounced than the stimulation achieved by MP (0.1μM), with and without addition of SEB (Fig 2C). Notwithstanding the inverse concentration gradients for MP and compound A, the addition of SEB remains able to significantly stimulate the IL-10 production even further. Exemplary, the condition treated with SEB and MP 0.1μM is significantly higher than the condition with MP 0.1μM alone (*P*<0.01) (Fig 2C). In inferior turbinate tissue the secreted levels of IL-10 hardly surpass threshold measurements. Nevertheless, the above-mentioned effects observed for PMBCs are also trending in this tissue, but do not reach overall significance (Fig 2D).

### The impact of compound A and methylprednisolone on IL-17

**Fig 3:**
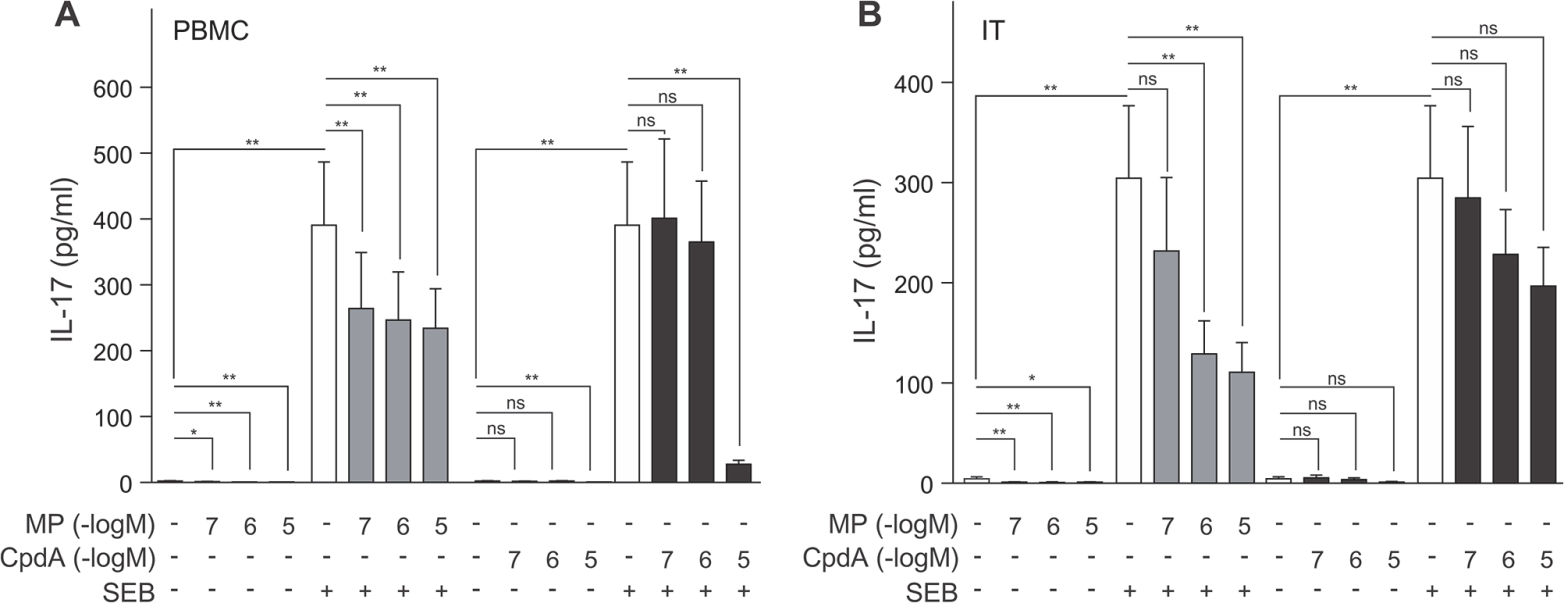
Methylprednisolone and compound A inhibit SEB-induced IL-17 production with a different and tissue-dependent sensitivity. *(A)* PBMC cells and *(B)* processed nasal inferior turbinate tissues (IT) were treated with methylprednisolone (MP) (0.1μM, 1μM or 10μM) or compound A (CpdA) (0.1μM, 1μM or 10μM) for 1h, followed by a 24h incubation with SEB (0.5μg/ml). Cell culture media were analyzed for the presence of IL-17. Averaged results of 10 (PBMC) or 9 (IT) patient samples are shown ± SEM. Statistical analysis was performed using a Wilcoxon matched-pairs signed-rank test to analyze significance of select condition to condition comparisons. ns, not significant; *, *P*<0.05; **, *P*<0.01.

Next, we branched out to assay the Th17 cytokine IL-17 and how it is impacted by MP and compound A in PBMCs and inferior turbinate tissue. As expected [29], SEB stimulation caused a significant increase in IL-17 secretion from both PBMCs and inferior turbinate tissue (Fig 3). Both compound A and MP can repress IL-17 production, albeit with a different pharmacological profile (Fig 3). In PBMCs, MP represses SEB-induced IL-17 from 0.1 μM MP onwards, while compound A can only significantly repress SEB-stimulated IL-17 production as of 10 μM compound A (Fig. 3A). In SEB-treated inferior turbinate tissue, only exposure to MP 1μM and MP 10μM can impose a significant repression on the IL-17 secretion (Fig 3B).

### The impact of compound A and methylprednisolone on pro-inflammatory cytokines

**Fig 4:**
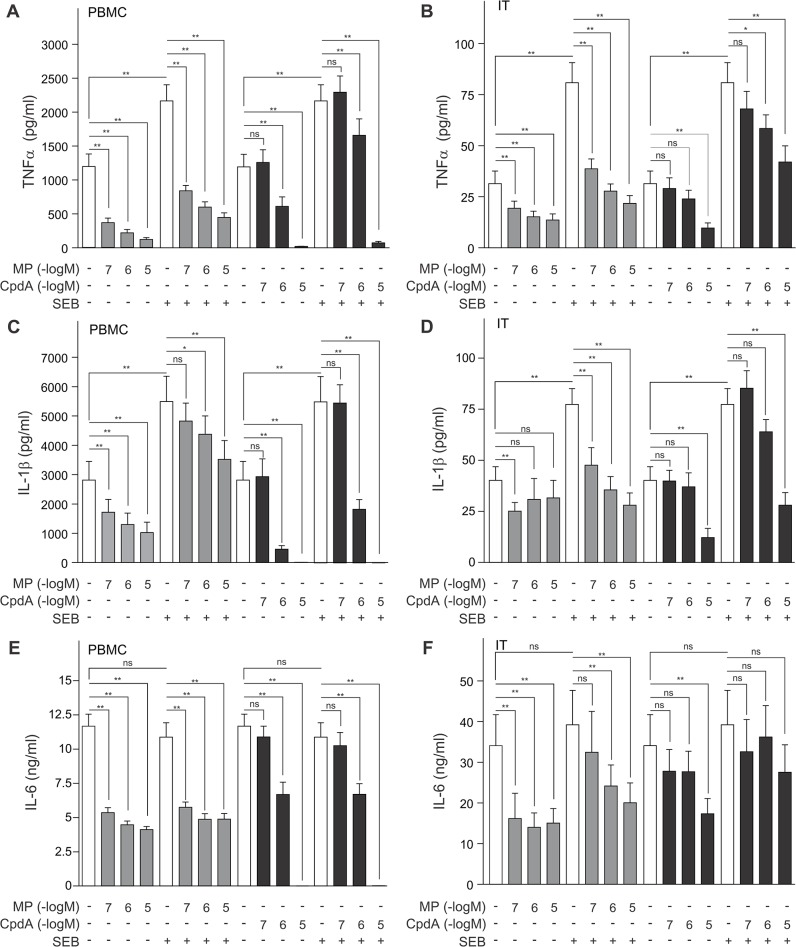
Methylprednisolone and compound A concentration-dependently inhibit TNFα, IL-1β and IL-6 production with a different and tissue-dependent sensitivity. *(A*,*C*,*E)* PBMC cells and *(B*,*D*,*F)* processed nasal inferior turbinate tissues (IT) were treated with methylprednisolone (MP) (0.1μM, 1μM or 10μM) or compound A (CpdA) (0.1μM, 1μM or 10μM) for 1h, followed by a 24h incubation with SEB (0.5μg/ml). Cell culture media were analyzed for the presence of TNFα *(A*,*B)*, IL-1β *(C*,*D)* or IL-6 *(E*,*F)*. Averaged results of 10 (PBMC) or 9 (IT) patient samples are shown ± SEM. Statistical analysis was performed using a Wilcoxon matched-pairs signed-rank test to analyze significance of select condition to condition comparisons. ns, not significant; *, *P*<0.05; **, *P*<0.01.

In the last panel of cytokine analyses, the effect of MP and compound A on the pro-inflammatory cytokines TNFα, IL-1β and IL-6 was assayed and we observed that these cytokines behave quite similarly. The secreted levels of both TNFα and IL-1β are significantly augmented in SEB-treated PBMCs and inferior turbinate tissue, when compared to controls (Fig 4). Furthermore, MP can significantly repress basal and SEB-stimulated TNFα and IL-1β production in a concentration-dependent manner, albeit less pronounced for IL-1β (Fig 4A–4D). Even so, compound A can inhibit basal and SEB-stimulated TNFα and IL1β production in a concentration-dependent manner, with a near to complete abrogation of cytokine production when PBMCs were treated with compound A at 10μM (Fig 4A and 4C). The TNFα and IL-1β production in inferior turbinate tissue was also concentration-dependently diminished by compound A (Fig 4B and 4D). In PBMCs, compound A and MP repress TNFα and IL-1β cytokine production well below basal control levels (Fig 4A and 4C).

As expected with the applied stimulus, both in PBMCs and inferior turbinate tissue, the production of IL-6 cannot be raised by the addition of SEB (Fig 4E and 4F). Nonetheless, MP, starting from 0.1μM in PBMCs and 1μM in inferior turbinate tissue, can significantly diminish IL-6 cytokine production (Fig 4E and 4F). Also, compound A, starting from 1μM, can inhibit IL-6 protein levels in PBMCs, with a complete abrogation of IL-6 production after exposure to compound A 10μM (Fig 4E), far beyond the baseline level that can be reached using MP. Compound A is, however, not able to repress IL-6 production in inferior turbinate tissue (Fig 4F).

### The selective GR modulator compound A does not affect cell viability of PBMCs

To complement our assessment of the effects of the non-steroidal selective GR modulator compound A in human cells and tissue, we set out to assay whether this compound could affect cell viability. The often spectacular drop in PBMC cytokine production associated with a 10μM compound A treatment could lead to suspect a possible effect of this selective GR modulator on cell survival.

**Fig 5:**
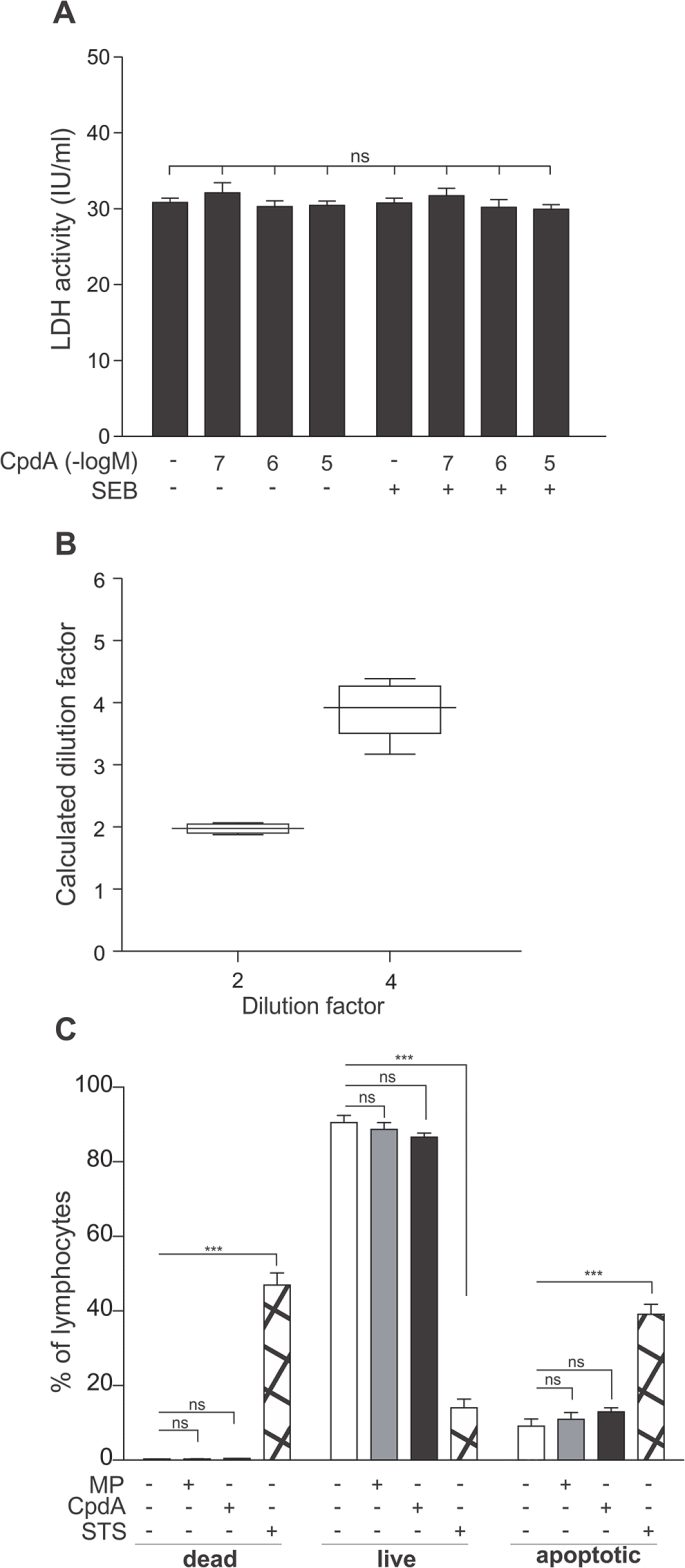
The selective GR modulator compound A does not affect cell viability of PBMCs. *(A)* PBMC cells were treated with compound A (CpdA) (0.1μM, 1μM or 10μM) for 1h, followed by a 24h incubation with SEB (0.5μg/ml). Cell culture media were analyzed for the presence of LDH. Averaged results of 10 patient samples are shown ± SEM. Statistical analysis was performed using a Friedman test to compare all samples. ns, not significant *(B)* Six selected samples were diluted 1:2 and 1:4 and measured for LDH activity. Results of the undiluted sample were set as 1 and results of diluted samples were recalculated accordingly to obtain the ‘calculated dilution factor’. The resulting calculated dilution factors are presented as a box and whiskers plot and graphed against the theoretically expected value, i.e. the dilution factor on the X-axis. *(C)* PBMCs of 6 patients were exposed to solvent, methylprednisolone (MP) (10μM) or compound A (CpdA) (10μM). PBMCs of 4 patients were exposed to staurosporine (STS) (10μM) for 24 h, as a positive control. Cell apoptosis and cell death was analyzed using flow cytometric analysis gated on the lymphocytes, of annexin V binding and propidium iodide staining, respectively. Averaged results are shown ± SEM. Statistical analysis was performed using a two-way ANOVA with Bonferroni post-tests to analyze the significance of treatments versus the solvent control. ns, not significant; **, *P*<0.01; ***, *P*<0.001.

To analyze this, we pretreated PMBCs with solvent or varying concentrations of compound A either or not followed by SEB, and analyzed the lactate dehydrogenase content of the medium. This oxidoreductase mediates the interconversion of lactate and pyruvate, and is released into the medium when membrane integrity is lost, thus acting as a measure for cell damage. The analysis of 10 PBMC patient samples showed no significant differences in LDH activity, and thus no significant differences in cell damage, between the various treatments and across the different patient samples (Fig 5A). A dilution test, measuring 1:2 and 1:4 dilutions of select samples, showed no statistically significant difference between our actual and our theoretically expected data, indicating that our observations are observed within the linear range (Fig 5B). In conclusion, compound A (0.1μM, 1μM or 10μM), either or not combined with SEB, does not impact PBMC cell membrane integrity.

Additionally, we assessed the binding of the phospholipid binding protein annexin V to potentially externalized phophatidylserine residues to the plasma membrane, as a hallmark for a cell undergoing apoptosis, using flow cytometry gated on the lymphocytes. Propidium iodide is used as a marker of cell death in this assay. We could show that both MP (10μM) and compound A (10μM) do not significantly impact the annexin V binding and thus induction of apoptosis in the PBMC lymphocytes (Fig 5C). A selection of PBMCs was also exposed to staurosporine for 24 h, as a positive control. Indeed, staurosporine (10μM) can significantly enhance annexin V binding and thus the number of cells showing apoptotic events in the PBMC lymphocyte fraction (Fig 5C).

### RU486 enforces the transrepressing activity of compound A, and selectively counteracts methylprednisolone’s repressing effects

Lastly, we performed an additional experiment with a different set of PMBCs using compound A and methylprednisolone and investigated whether their activity could be abrogated or counteracted by the GR and progesterone receptor inhibitor RU486 (also known as mifepristone) [30,31]. The exact binding mode of compound A on GR remains unresolved; it possibly binds (differently) within the ligand-binding pocket or not even in the ligand-binding pocket at all. Additionally, only a partial glucocorticoid displacement can be attained using increasing competition with compound A [21]. Moreover, RU486 on its own can also act as a partial agonist in both transactivation and transrepression in some cells [32–36]. It may thus potentially partially stimulate the expression of GILZ and DUSP1 in PBMCs and display its own partial transrepressing actions on cytokines, and may thus perturb the interpretation of compound A and RU486 combination experiments even further. Notwithstanding the evidence clouding the mechanistic interpretation of RU486-based experiments, RU486 is a clinically approved drug and we were interested to investigate its effects on a methylprednisolone- or compound A-mediated regulation of cytokines.

**Fig 6:**
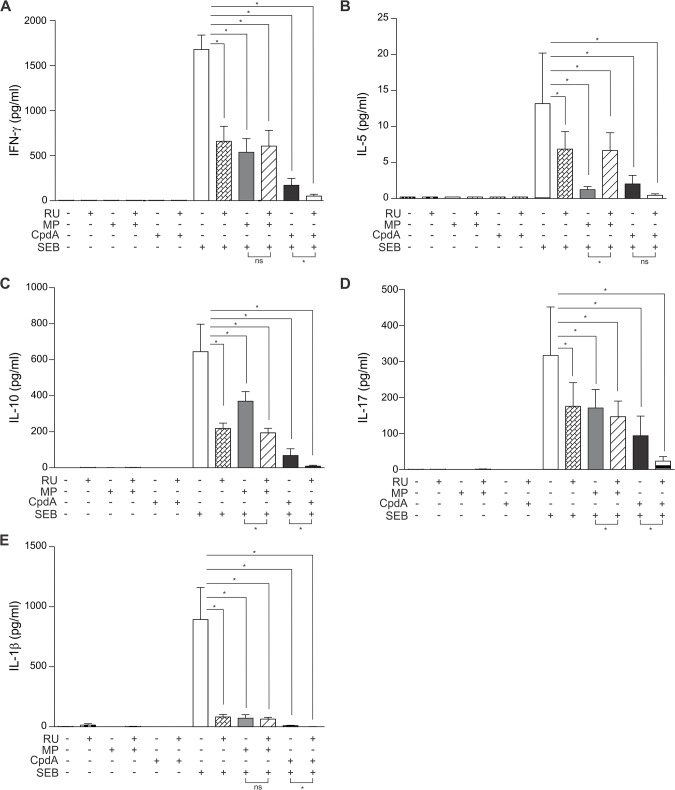
RU486 enforces the transrepressing activity of compound A, and selectively counteracts methylprednisolone’s repressing effects. PBMC cells were pretreated with solvent or RU486 (RU) (20μM) for 30 minutes, followed by a treatment with solvent, methylprednisolone (MP) (1μM) or compound A (CpdA) (10μM) for 1h, either or not ensued by a 24h incubation with SEB (0.5μg/ml). Cell culture media were analyzed for the presence of IFN-γ *(A)*, IL-5 *(B)*, IL-10 *(C)*, IL-17 *(D)*, IL-1β *(E)*. Averaged results of 6 patient samples are shown ± SEM. Statistical analysis was performed using a Wilcoxon matched-pairs signed-rank test to analyze significance of select condition to condition comparisons. ns, not significant; *, *P*<0.05.

As expected, we could show that RU486 can indeed act as a partial GR agonist in transrepression, by itself partially repressing the secretion of monitored cytokines (Fig 6A–6E). Although RU486 was functional in counteracting a classic GRE-regulated gene, namely GILZ, at 2μM [37], RU486 at a 10 fold higher concentration was only able to counteract methylprednisolone-mediated repression of IL-5 (Fig 6B), and not of the other monitored cytokines in PBMCs (Fig 6A and 6C–6E). Likely due to the evidence mentioned above, RU486 could not reverse compound A-mediated effects on SEB-stimulated PBMC-secreted cytokines. In fact, the partial agonistic properties of RU486 even add on to the compound A-mediated repression of IFNγ, IL-10, IL-17 and IL-1β(Fig 6A and 6C–6E), suggesting that compound A either binds outside of the GR ligand-binding pocket or acts independently of GR itself. Note that these results using a different set of patient samples show different sensitivities for SEB onto IL-10 and for MP onto IL-10, IFN-γ and IL-1β, suggesting a patient-specific sensitivity towards GR responses and the inflammatory stimulus.

## Discussion

In this study we investigated the effects of classic glucocorticoids *versus* the effects of compound A on the ability of peripheral blood mononuclear cells (PBMCs) (Table 1) as well as inferior turbinate tissue (Table 2) to respond to a challenge with *Staphylococcus aureus*–derived enterotoxin B protein (SEB), previously used in an established model to investigate human nasal polyposis. We could show that both compound A and the tested glucocorticoid can inhibit cytokine release and augment the production of the inhibitory cytokine IL-10, albeit with compound-specific amplitudes.

**Table 1:** Summary of the significant effects of the selective GR modulator compound A and the glucocorticoid methylprednisolone on PBMCs.

	**SEB** ^a^	**MP** ^b^ **/SEB**		**CpdA** ^c^ **/SEB**	
	**Direction**	**Direction**	**Concentration**	**Direction**	**Concentration**
**IL** ^**d**^ **-2**	↗	↘	0.1 μM	↘	10 μM
**IFN** ^**e**^ **-γ**	↗	↘	10 μM	↗	0.1 μM
				↘	10 μM
**IL-5**	↗	↘	0.1 μM	↘	10 μM
**IL-10**	Ns	↗	0.1 μM	↗	0.1 μM
		↘	10 μM	↘	10 μM
**IL-17**	↗	↘	0.1 μM	↘	10 μM
**TNF** ^**f**^ **-α**	↗	↘	0.1 μM	↘	1 μM
**IL-1β**	↗	↘	1 μM	↘	1 μM
**IL-6**	ns	↘	0.1 μM	↘	1 μM

^a^ SEB, *Staphylococcus aureus*–derived enterotoxin B protein

^b^ MP, methylprednisolone

^c^ CpdA, compound A

^d^ IL, interleukin

^e^ IFN, interferon

^f^ TNF, tumor necrosis factor

A difference was considered significant as of *P*<0.05 and its directionality is indicated with upward (↗) or downward (↘) arrows. For the concentration ranges of methylprednisolone and compound A, we also provide the minimal concentration to achieve the respective significant effect.

**Table 2:** Summary of the significant effects of the selective GR modulator compound A and the glucocorticoid methylprednisolone on inferior turbinate tissue.

	**SEB** ^a^	**MP** ^b^ **/SEB**		**CpdA** ^c^ **/SEB**	
	**Direction**	**Direction**	**Concentration**	**Direction**	**Concentration**
**IL** ^**d**^ **-2**	↗	↘	1 μM	ns	
**IFN** ^**e**^ **-γ**	ns	↗	0.1 μM	↗	0.1 μM
		↘	10 μM		
**IL-5**	ns	ns		ns	
**IL-10**	ns	ns		↗	0.1 μM
**IL-17**	↗	↘	1 μM	ns	
**TNF** ^**f**^ **-α**	↗	↘	0.1 μM	↘	1 μM
**IL-1β**	↗	↘	0.1 μM	↘	10 μM
**IL-6**	ns	↘	1 μM	ns	

^a^ SEB, *Staphylococcus aureus*–derived enterotoxin B protein

^b^ MP, methylprednisolone

^c^ CpdA, compound A

^d^ IL, interleukin

^e^ IFN, interferon

^f^ TNF, tumor necrosis factor

A difference was considered significant as of *P*<0.05 and its directionality is indicated with upward (↗) or downward (↘) arrows. For the concentration ranges of methylprednisolone and compound A, we also provide the minimal concentration to achieve the respective significant effect.

Although compound A has known cytotoxic effects in a selected variety of immortalized cancer cell lines [38,39], cell membrane instability LDH analyses of compound A-exposed PBMCs clearly showed an absence of cell death events in these PBMCs (Fig 5A). Furthermore, neither compound A nor methylprednisolone could induce apoptosis-indicating annexin V binding in lymphocytes (Fig 5C). We already knew that glucocorticoids did not have a profound effect on cell viability, as a previous report using transmission electron microscopy, already demonstrated that PBMCs left untreated or treated with glucocorticoids, in this case dexamethasone, died at similar rates over a course of 48h [40].

In the current study, all PBMC-secreted cytokines, save IL-6 and IL-10, were significantly upregulated by SEB stimulation (Table 1 and Figs 1–4), which concurs with current publications on SEB-induced cytokine production of IL-2, IFN-γ, IL-5, IL-17, TNFα and IL1-β [41,42]. The inferior turbinate tissue of healthy subjects displayed only a SEB-stimulated significant increase for the cytokines IL-2, IL-17, TNFα and IL-1β (Figs 1, 3 and 4), whilst also IFN-γ and IL-10 have been reported to significantly increase in this tissue after SEB exposure [27]. Similarly, nasal polyp tissue displays a SEB-stimulated significant release of IL-1, TNFα, IFN-γ, IL-2, IL-5 and IL-17 [28]. As enterotoxins act as superantigens via polyclonal T cell activation [43], the lack of a SEB-mediated stimulus on IL-6 production, mainly by monocytes, was to be expected (Fig 4E and 4F). A remarkable tissue-dependent response difference does occur for IFN-γ. In PBMCs, SEB induces an 8-fold increase in IFN-γ production, whereas in inferior turbinate tissue the SEB-stimulated IFN-γ hardly surpasses the detection threshold, even after stimulation. Overall, our data indicate that SEB is capable of stimulating all prominent T helper cell populations.

Th1, Th2 and also Th17 cells were reported to be implicated in chronic diseases of the paranasal sinuses, Th1-related cytokine IFN-*γ* and Th2-related cytokines IL-4 and IL-5 in chronic rhinosinusitis without and with nasal polyps, respectively, and more recently Th17-cell-related IL-17 in nasal polyps [44–46]. Hence, to effectively tackle inflammation, one requires a drug affecting a wide range of activities in different T cell populations. Exposure to glucocorticoids in the early activation phase of T-cells procures an inhibition of IL-2 and IFN-γ production, whilst stimulating the cytokine IL-10, expected to inhibit a Th1 response. In acute treatment regimens however, the production of both IL-4 and IL-5 is also inhibited by glucocorticoids, as such impeding a Th2 response. In analogy, compound A has been shown to inhibit the OVA-induced IL-4 and IL-5 Th2 cytokine production in bronchoalveolar lavage fluid in a murine model of OVA-induced asthma [47]. Taken together, the clinical applicability of glucocorticoids expands from auto-immune disease to the treatment of asthma and allergies [48,49]. The myriad of glucocorticoid effects in immune cells and disorders is also clearly affected by a cross talk between cytokines and glucocorticoid action [50,51], with cytokines negatively affecting the activity of the glucocorticoid receptor. Although intricate and currently incompletely resolved, researchers have started addressing this conundrum already many decades ago. However, for compound A the picture is currently far less clear.

From *in vivo* murine and *in vitro* experiments using compound A, we know that this selective GR modulator actively favors the formation of GR monomers and as such precludes classic GRE stimulation of side-effect associated genes, but also of anti-inflammatory genes such as GILZ. Similar to classic glucocorticoids, compound A has NF-κB-dependent anti-inflammatory properties exerted by inhibiting pro-inflammatory gene expression [21,22, 23, 52]. Here, we show that both compound A and the glucocorticoid methylprednisolone are capable of repressing IL-2, IFN-γ, IL-5, IL-6, IL-10, IL-17, IL-1β and TNFα expression in human PBMCs (Table 1 and Figs 1–4), suggestive of a general anti-inflammatory action profile. In the inferior turbinate tissue, treatment with methylprednisolone could significantly repress IL-2, IFN-γ, IL-6, IL-17, TNFα and IL-1β production, while a compound A treatment only allowed for a significant repression of IL-1β and TNFα (Table 2 and Figs 1–4). Overall, the compound A-induced repression window of cytokines showed to be far greater in PBMCs than in inferior turbinate tissue. However, this could be explained as in our results PBMCs generally express higher cytokine levels than samples of inferior turbinate tissue, except for IL-6, in which the inferior turbinate tissue levels exceed PBMC levels, and IL-17, which appears to be produced in a similar range in both experimental settings. Furthermore, we noticed the resemblance in the IL-2, and IL-17 responses to compound A in PBMCs, which did not show any effect for compound A 1μM, while displaying a clearly abrogated cytokine production after exposure to compound A 10μM (Fig 1 and 3). Although we report a slightly more sensitive and more gradual response profile for IL-6, TNFα and IL-1β (Fig 4), all cytokines showed a steep decline to near or below baseline levels at compound A 10μM.

Interestingly, both IFN-γ and IL-10 seem to respond differently to methylprednisolone and compound A, depending on the administered concentration. Low concentrations of the selective GR modulator compound A result in a surge of IFN-γ and IL-10 levels, while high concentrations of compound A actually result in a decrease in IFN-γ and IL-10 levels in PBMCs (Figs 1C and 2C).When using the classic glucocorticoid methylprednisolone, this response profile is reiterated, albeit in a milder form (Figs 1C and 2C). Of note, IFN-γ was previously shown to be able to inhibit Th2 cytokine production and IL-10 can inhibit a Th1 response [48]. Moreover, IL-10 can act as a sensitizer for glucocorticoid responsiveness [51].

Although compound A is unable to stimulate classic GRE-regulated gene transcription [21], recent publications on compound A-stimulated gene expression of Hsp70 [53] and on compound A-mediated stimulation of the transcription factor GATA-3 [54], together with our current report on a compound A-mediated increase in IFN-γ and IL-10 production, support the notion that compound A can stimulate gene transcription via alternate mechanisms. Often, but not always, these mechanisms are also utilized by classic glucocorticoids and the GR. Given the fact that compound A has shown both GR-dependent and GR-independent mechanisms in previous reports [21,47,52,55,56], we cannot exclude compound A-mediated GR-independent effects. However, we wish to emphasize that all compound A-mediated effects on cytokine and chemokine repression thus far have shown to be mediated via GR, using full GR knockdown or knockout approaches [21,47,52,55]. Combining this insight with the additive repressive effect of RU486 and compound A on PBMC cytokine levels, suggests that compound A represses cytokine levels via a GR-mediated mechanism, but may either bind within or outside the ligand-binding pocket of GR in a differential manner. In support, compound A induces a different, currently unclarified, conformational change of GR [21]. Although *in silico* modeling mapped compound A to fit the ligand-binding pocket of GR [39], other modes of binding cannot be excluded, because we still await the first elucidated crystal structure of this particular selective GR modulator binding to the GR ligand-binding domain.

Furthermore, the concept of glucocorticoid concentration-dependent effects on gene expression have been noticed previously in other settings, but remains often unexplained [57–59]. Although pharmacological response profile analyses are commonly performed using a range of concentrations, mechanistic studies are still quite often limited to one concentration. For instance, in murine T-cells it was earlier reported that a 10μM concentration of compound A can diminish IFN-γ levels via an inhibition of the transcription factor T-bet [54]. However, the authors did not investigate the effect of lower concentrations of compound A on the Th1 cytokine IFN-γ. Nevertheless, their results do suggest that a low concentration compound A-mediated upregulation of IFN-γ, if any in this system, would probably not stem from a stimulation of T-bet [54]. A species-specific event on PBMC cannot be excluded, as *in vivo* compound A-treated murine PBMCs also show diminished IFN-γ levels [60]. In conclusion, the interplay of Th1 and Th2 immunity under the influence of a selective GR modulator deserves further investigation.

In conclusion, both the glucocorticoid methylprednisolone, and the novel selective GR modulator compound A display anti-inflammatory actions in both ex vivo PBMC and a nasal tissue stimulation model of nasal polyposis. Combining compound A ‘s established improved side effect profile pertaining to bone and glucose metabolism together with our current results, allows to advise further research into a novel generation of more stabile selective GR modulators as a new anti-inflammatory therapy in clinic to evaluate their therapeutic benefit.

## References

[1] Hayden MS, Ghosh S (2008) Shared principles in NF-kappaB signaling. Cell 132: 344–362. 10.1016/j.cell.2008.01.020 18267068

[2] Hayden MS, Ghosh S (2012) NF-kappaB, the first quarter-century: remarkable progress and outstanding questions. Genes Dev 26: 203–234. 10.1101/gad.183434.111 22302935 PMC3278889

[3] Bhatt D, Ghosh S (2014) Regulation of the NF-kappaB-Mediated Transcription of Inflammatory Genes. Front Immunol 5: 71 10.3389/fimmu.2014.00071 24611065 PMC3933792

[4] Vanden Berghe W, Vermeulen L, De Wilde G, De Bosscher K, Boone E, Haegeman G (2000) Signal transduction by tumor necrosis factor and gene regulation of the inflammatory cytokine interleukin-6. Biochem Pharmacol 60: 1185–1195. 11007957 10.1016/s0006-2952(00)00412-3

[5] Barnes PJ, Chung KF, Page CP (1998) Inflammatory mediators of asthma: an update. Pharmacol Rev 50: 515–596. 9860804

[6] Bachert C, van Cauwenberge P, Olbrecht J, van Schoor J (2006) Prevalence, classification and perception of allergic and nonallergic rhinitis in Belgium. Allergy 61: 693–698. 16677237 10.1111/j.1398-9995.2006.01054.x

[7] Hastan D, Fokkens WJ, Bachert C, Newson RB, Bislimovska J, Bockelbrink A, et al (2011) Chronic rhinosinusitis in Europe—an underestimated disease. A GA(2)LEN study. Allergy 66: 1216–1223. 10.1111/j.1398-9995.2011.02646.x 21605125

[8] Bousquet J, Bieber T, Fokkens W, Humbert M, Kowalski ML, Niggemann B, et al (2008) Consensus statements, evidence-based medicine and guidelines in allergic diseases. Allergy 63: 1–4. 10.1111/j.1398-9995.2008.01897.x 18053012

[9] Fokkens W (2007) Role of steroids in the treatment of rhinosinusitis with and without polyposis. Clin Allergy Immunol 20: 241–250. 17534055

[10] Villa E, Magnoni MS, Micheli D, Canonica GW (2011) A review of the use of fluticasone furoate since its launch. Expert Opin Pharmacother 12: 2107–2117. 10.1517/14656566.2011.600688 21797803

[11] Passalacqua G, Albano M, Canonica GW, Bachert C, Van Cauwenberge P, Davies RJ, et al (2000) Inhaled and nasal corticosteroids: safety aspects. Allergy 55: 16–33. 10696853 10.1034/j.1398-9995.2000.00370.x

[12] Bachert C, Hormann K, Mosges R, Rasp G, Riechelmann H, Müller R, et al (2003) An update on the diagnosis and treatment of sinusitis and nasal polyposis. Allergy 58: 176–191. 12653791 10.1034/j.1398-9995.2003.02172.x

[13] Beck IM, Vanden Berghe W, Vermeulen L, Yamamoto KR, Haegeman G, De Bosscher K. (2009) Crosstalk in inflammation: the interplay of glucocorticoid receptor-based mechanisms and kinases and phosphatases. Endocr Rev 30: 830–882. 10.1210/er.2009-0013 19890091 PMC2818158

[14] Ratman D, Vanden Berghe W, Dejager L, Libert C, Tavernier J, Beck IM, et al (2013) How glucocorticoid receptors modulate the activity of other transcription factors: a scope beyond tethering. Mol Cell Endocrinol 380: 41–54. 10.1016/j.mce.2012.12.014 23267834

[15] Surjit M, Ganti KP, Mukherji A, Ye T, Hua G, Metzger D, et al (2011) Widespread negative response elements mediate direct repression by agonist-liganded glucocorticoid receptor. Cell 145: 224–241. 10.1016/j.cell.2011.03.027 21496643

[16] Pedersen S (1999) Comparing inhaled glucocorticosteroids. Allergy 54 Suppl 49: 42–50. 10422747 10.1111/j.1398-9995.1999.tb04387.x

[17] Schacke H, Docke WD, Asadullah K (2002) Mechanisms involved in the side effects of glucocorticoids. Pharmacol Ther 96: 23–43. 12441176 10.1016/s0163-7258(02)00297-8

[18] McDonough AK, Curtis JR, Saag KG (2008) The epidemiology of glucocorticoid-associated adverse events. Curr Opin Rheumatol 20: 131–137. 10.1097/BOR.0b013e3282f51031 18349741

[19] Pujols L, Mullol J, Torrego A, Picado C (2004) Glucocorticoid receptors in human airways. Allergy 59: 1042–1052. 15355461 10.1111/j.1398-9995.2004.00635.x

[20] Louw A, Swart P, de Kock SS, van der Merwe KJ (1997) Mechanism for the stabilization in vivo of the aziridine precursor—(4-acetoxyphenyl)-2-chloro-N-methyl-ethylammonium chloride by serum proteins. Biochem Pharmacol 53: 189–197. 9037251 10.1016/s0006-2952(96)00661-2

[21] De Bosscher K, Vanden Berghe W, Beck IM, Van Molle W, Hennuyer N, Hapgood J, et al (2005) A fully dissociated compound of plant origin for inflammatory gene repression. Proc Natl Acad Sci U S A 102: 15827–15832. 16243974 10.1073/pnas.0505554102 PMC1276063

[22] Dewint P, Gossye V, De Bosscher K, Vanden Berghe W, Van Beneden K, Deforce D, et al (2008) A plant-derived ligand favoring monomeric glucocorticoid receptor conformation with impaired transactivation potential attenuates collagen-induced arthritis. J Immunol 180: 2608–2615. 18250472 10.4049/jimmunol.180.4.2608

[23] De Bosscher K, Beck IM, Haegeman G (2010) Classic glucocorticoids versus non-steroidal glucocorticoid receptor modulators: survival of the fittest regulator of the immune system? Brain Behav Immun 24: 1035–1042. 10.1016/j.bbi.2010.06.010 20600811

[24] van Loo G, Sze M, Bougarne N, Praet J, Mc Guire C, Ullrich A, et al (2010) Antiinflammatory properties of a plant-derived nonsteroidal, dissociated glucocorticoid receptor modulator in experimental autoimmune encephalomyelitis. Mol Endocrinol 24: 310–322. 10.1210/me.2009-0236 19965930 PMC5428123

[25] Zhang Z, Zhang ZY, Schluesener HJ (2009) Compound A, a plant origin ligand of glucocorticoid receptors, increases regulatory T cells and M2 macrophages to attenuate experimental autoimmune neuritis with reduced side effects. J Immunol 183: 3081–3091. 10.4049/jimmunol.0901088 19675162

[26] Robertson S, Allie-Reid F, Berghe WV, Visser K, Binder A, Africander D, et al (2010) Abrogation of glucocorticoid receptor dimerization correlates with dissociated glucocorticoid behavior of compound A. J Biol Chem 285: 8061–8075. 10.1074/jbc.M109.087866 20037160 PMC2832957

[27] Patou J, Gevaert P, Van Zele T, Holtappels G, van Cauwenberge P, Bachert C (2008) Staphylococcus aureus enterotoxin B, protein A, and lipoteichoic acid stimulations in nasal polyps. J Allergy Clin Immunol 121: 110–115. 17980412 10.1016/j.jaci.2007.08.059

[28] Zhang N, Van Crombruggen K, Holtappels G, Bachert C (2012) A Herbal Composition of Scutellaria baicalensis and Eleutherococcus senticosus Shows Potent Anti-Inflammatory Effects in an Ex Vivo Human Mucosal Tissue Model. Evid Based Complement Alternat Med 2012: 673145. 10.1155/2012/673145 PMC3261630 22272213

[29] Islander U, Andersson A, Lindberg E, Adlerberth I, Wold AE, Rudin A (2010) Superantigenic Staphylococcus aureus stimulates production of interleukin-17 from memory but not naive T cells. Infect Immun 78: 381–386. 10.1128/IAI.00724-09 19822653 PMC2798201

[30] Gompel A, Malet C, Spritzer P, Lalardrie JP, Kuttenn F, Mauvais-Jarvis P (1986) Progestin effect on cell proliferation and 17 beta-hydroxysteroid dehydrogenase activity in normal human breast cells in culture. J Clin Endocrinol Metab 63: 1174–1180. 2428825 10.1210/jcem-63-5-1174

[31] Bigsby RM, Young PC (1993) Progesterone and dexamethasone inhibition of uterine epithelial cell proliferation: studies with antiprogesterone compounds in the neonatal mouse. J Steroid Biochem Mol Biol 46: 253–257. 8664174 10.1016/0960-0760(93)90301-c

[32] Hadley KE, Louw A, Hapgood JP (2011) Differential nuclear localisation and promoter occupancy play a role in glucocorticoid receptor ligand-specific transcriptional responses. Steroids 76: 1176–1184. 10.1016/j.steroids.2011.05.007 21641918

[33] Ronacher K, Hadley K, Avenant C, Stubsrud E, Simons SS Jr., Louw A, et al (2009) Ligand-selective transactivation and transrepression via the glucocorticoid receptor: role of cofactor interaction. Mol Cell Endocrinol 299: 219–231. 10.1016/j.mce.2008.10.008 19007848

[34] Robertson S, Rohwer JM, Hapgood JP, Louw A (2013) Impact of glucocorticoid receptor density on ligand-independent dimerization, cooperative ligand-binding and basal priming of transactivation: a cell culture model. PLoS One 8: e64831 10.1371/journal.pone.0064831 23717665 PMC3661511

[35] Schulz M, Eggert M, Baniahmad A, Dostert A, Heinzel T, Renkawitz R (2002) RU486-induced glucocorticoid receptor agonism is controlled by the receptor N terminus and by corepressor binding. J Biol Chem 277: 26238–26243. 12011091 10.1074/jbc.M203268200

[36] Schoch GA, D'Arcy B, Stihle M, Burger D, Bär D, Benz J, et al (2010) Molecular switch in the glucocorticoid receptor: active and passive antagonist conformations. J Mol Biol 395: 568–577. 10.1016/j.jmb.2009.11.011 19913032

[37] Drebert Z, Bracke M, Beck IM (2015) Glucocorticoids and the non-steroidal selective glucocorticoid receptor modulator, compound A, differentially affect colon cancer-derived myofibroblasts. J Steroid Biochem Mol Biol. 10.1016/j.jsbmb.2015.02.002 25666906

[38] Lesovaya EA, Yemelyanov AY, Kirsanov KI, Yakubovskaya MG, Budunova IV (2011) Antitumor effect of non-steroid glucocorticoid receptor ligand CpdA on leukemia cell lines CEM and K562. Biochemistry (Mosc) 76: 1242–1252. 10.1134/S000629791111006X 22117551

[39] Yemelyanov A, Czwornog J, Gera L, Joshi S, Chatterton RT Jr., Budunova I (2008) Novel steroid receptor phyto-modulator compound a inhibits growth and survival of prostate cancer cells. Cancer Res 68: 4763–4773. 10.1158/0008-5472.CAN-07-6104 18559523

[40] Totino PR, Riccio EK, Corte-Real S, Daniel-Ribeiro CT, de Fatima Ferreira-da-Cruz M (2006) Dexamethasone has pro-apoptotic effects on non-activated fresh peripheral blood mononuclear cells. Cell Biol Int 30: 133–137. 16271306 10.1016/j.cellbi.2005.09.002

[41] Krakauer T (1995) Differential inhibitory effects of interleukin-10, interleukin-4, and dexamethasone on staphylococcal enterotoxin-induced cytokine production and T cell activation. J Leukoc Biol 57: 450–454. 7884317 10.1002/jlb.57.3.450

[42] Perez Novo CA, Jedrzejczak-Czechowicz M, Lewandowska-Polak A, Claeys C, Holtappels G, Van Cauwenberge P, et al (2010) T cell inflammatory response, Foxp3 and TNFRS18-L regulation of peripheral blood mononuclear cells from patients with nasal polyps-asthma after staphylococcal superantigen stimulation. Clin Exp Allergy 40: 1323–1332. 10.1111/j.1365-2222.2010.03577.x 20701615

[43] Bachert C, Zhang N, Patou J, van Zele T, Gevaert P (2008) Role of staphylococcal superantigens in upper airway disease. Curr Opin Allergy Clin Immunol 8: 34–38. 10.1097/ACI.0b013e3282f4178f 18188015

[44] Van Zele T, Claeys S, Gevaert P, Van Maele G, Holtappels G, Van Cauwenberge P, et al (2006) Differentiation of chronic sinus diseases by measurement of inflammatory mediators. Allergy 61: 1280–1289. 17002703 10.1111/j.1398-9995.2006.01225.x

[45] Zhang N, Van Zele T, Perez-Novo C, Van Bruaene N, Holtappels G, DeRuyck N, et al (2008) Different types of T-effector cells orchestrate mucosal inflammation in chronic sinus disease. J Allergy Clin Immunol 122: 961–968. 10.1016/j.jaci.2008.07.008 18804271

[46] Moon IJ, Hong SL, Kim DY, Lee CH, Rhee CS, Min YG (2012) Blocking interleukin-17 attenuates enhanced inflammation by staphylococcal enterotoxin B in murine allergic rhinitis model. Acta Otolaryngol 132 Suppl 1: S6–12. 10.3109/00016489.2012.661074 22582785

[47] Reber LL, Daubeuf F, Plantinga M, De Cauwer L, Gerlo S, Waelput W, et al (2012) A dissociated glucocorticoid receptor modulator reduces airway hyperresponsiveness and inflammation in a mouse model of asthma. J Immunol 188: 3478–3487. 10.4049/jimmunol.1004227 22393156

[48] Liberman AC, Druker J, Garcia FA, Holsboer F, Arzt E (2009) Intracellular molecular signaling. Basis for specificity to glucocorticoid anti-inflammatory actions. Ann N Y Acad Sci 1153: 6–13. 10.1111/j.1749-6632.2008.03958.x 19236322

[49] Zhang N, Van Crombruggen K, Holtappels G, Lan F, Katotomichelakis M, Zhang L, et al (2014) Suppression of cytokine release by fluticasone furoate vs. mometasone furoate in human nasal tissue ex-vivo. PLoS One 9: e93754 10.1371/journal.pone.0093754 24710117 PMC3977874

[50] Dejager L, Vandevyver S, Petta I, Libert C (2014) Dominance of the strongest: inflammatory cytokines versus glucocorticoids. Cytokine Growth Factor Rev 25: 21–33. 10.1016/j.cytogfr.2013.12.006 24412262

[51] Creed TJ, Lee RW, Newcomb PV, di Mambro AJ, Raju M, Dayan CM (2009) The effects of cytokines on suppression of lymphocyte proliferation by dexamethasone. J Immunol 183: 164–171. 10.4049/jimmunol.0801998 19542427

[52] De Bosscher K, Beck IM, Dejager L, Bougarne N, Gaigneaux A, Chateauvieux S, et al (2014) Selective modulation of the glucocorticoid receptor can distinguish between transrepression of NF-kappaB and AP-1. Cell Mol Life Sci 71: 143–163. 10.1007/s00018-013-1367-4 23784308 PMC3889831

[53] Beck IM, Drebert ZJ, Hoya-Arias R, Bahar AA, Devos M, Clarisse D, et al (2013) Compound A, a selective glucocorticoid receptor modulator, enhances heat shock protein Hsp70 gene promoter activation. PLoS One 8: e69115 10.1371/journal.pone.0069115 23935933 PMC3728325

[54] Liberman AC, Antunica-Noguerol M, Ferraz-de-Paula V, Palermo-Neto J, Castro CN, Druker J, et al (2012) Compound A, a dissociated glucocorticoid receptor modulator, inhibits T-bet (Th1) and induces GATA-3 (Th2) activity in immune cells. PLoS One 7: e35155 10.1371/journal.pone.0035155 22496903 PMC3322149

[55] Wust S, Tischner D, John M, Tuckermann JP, Menzfeld C, Hanisch UK, et al (2009) Therapeutic and adverse effects of a non-steroidal glucocorticoid receptor ligand in a mouse model of multiple sclerosis. PLoS One 4: e8202 10.1371/journal.pone.0008202 19997594 PMC2781169

[56] Gossye V, Elewaut D, Bougarne N, Bracke D, Van Calenbergh S, Haegeman G et al (2009) Differential mechanism of NF-kappaB inhibition by two glucocorticoid receptor modulators in rheumatoid arthritis synovial fibroblasts. Arthritis Rheum 60: 3241–3250. 10.1002/art.24963 19877072

[57] Beck IM, Clarisse D, Bougarne N, Okret S, Haegeman G, De Bosscher K (2013) Mitogen- and stress-activated protein kinase 1 MSK1 regulates glucocorticoid response element promoter activity in a glucocorticoid concentration-dependent manner. Eur J Pharmacol 715: 1–9. 10.1016/j.ejphar.2013.06.032 23831393

[58] Fürst R, Schroeder T, Eilken HM, Bubik MF, Kiemer AK, Zahler S, et al (2007) MAPK phosphatase-1 represents a novel anti-inflammatory target of glucocorticoids in the human endothelium. FASEB J 21: 74–80. 17099067 10.1096/fj.06-6752com

[59] Reddy TE, Pauli F, Sprouse RO, Neff NF, Newberry KM, Garabedian MJ, et al (2009) Genomic determination of the glucocorticoid response reveals unexpected mechanisms of gene regulation. Genome Res 19: 2163–2171. 10.1101/gr.097022.109 19801529 PMC2792167

[60] Rauner M, Thiele S, Sinningen K, Winzer M, Salbach-Hirsch J, Gloe I, et al (2013) Effects of the selective glucocorticoid receptor modulator compound A on bone metabolism and inflammation in male mice with collagen-induced arthritis. Endocrinology 154: 3719–3728. 10.1210/en.2012-2221 23885015

